# Olive Oil-Based Lipid Coating as a Precursor Organogel for Postharvest Preservation of Lychee: Efficacy Combined with Polyamide/Polyethylene Packaging Under Passive Atmosphere

**DOI:** 10.3390/gels11080608

**Published:** 2025-08-02

**Authors:** Alessandra Culmone, Roberta Passafiume, Pasquale Roppolo, Ilenia Tinebra, Vincenzo Naselli, Alfonso Collura, Antonino Pirrone, Luigi Botta, Alessandra Carrubba, Nicola Francesca, Raimondo Gaglio, Vittorio Farina

**Affiliations:** 1 Department of Agricultural, Food and Forest Sciences (SAAF), University of Palermo, Viale delle Scienze, 90128 Palermo, Italy; alessandra.culmone@unipa.it (A.C.); pasquale.roppolo@unipa.it (P.R.); ilenia.tinebra@unipa.it (I.T.); vincenzo.naselli@unipa.it (V.N.); antonino.pirrone@unipa.it (A.P.); alessandra.carrubba@unipa.it (A.C.); nicola.francesca@unipa.it (N.F.); raimondo.gaglio@unipa.it (R.G.); vittorio.farina@unipa.it (V.F.); 2 Bioagrifutura S.r.l., Via Ferruccio 2, Sant’Agata di Militello, 98076 Messina, Italy; alfonso.collura@inaf.it; 3 Istituto Nazionale di Astrofisica, Osservatorio Astronomico di Palermo, via G.F. Ingrassia 31, 90123 Palermo, Italy; 4 Department of Engineering, University of Palermo, Viale delle Scienze, Ed. 6, 90128 Palermo, Italy; luigi.botta@unipa.it; 5 Centre for Sustainability and Ecological Transition, University of Palermo, 90133 Palermo, Italy

**Keywords:** olive oil-based coating, structured lipid system, gel-like materials, postharvest preservation, passive modified atmosphere, organogel precursor

## Abstract

Lychee (*Lychee chinensis* Sonn.) is a tropical fruit highly appreciated for its vivid red color, sweet flavor, and nutritional properties. However, it is highly perishable, with postharvest losses often due to oxidative browning and dehydration. This study evaluated the organic olive oil coating (OC), a natural lipidic system with the potential to act as a precursor for organogel development, combined with polyamide/polyethylene (PA/PE) packaging under passive modified atmosphere. Fruits were harvested at commercial maturity and divided into two groups: OC-treated and untreated control (CTR). Both groups were stored at 5 ± 1 °C and 90 ± 5% relative humidity and analyzed on days 0, 3, 6, and 9. The OC-treated fruits showed significantly better retention of physical, chemical, microbiological, and sensory qualities. The coating reduced oxidative stress and enzymatic browning, preserving color and firmness. The PA/PE packaging regulated gas exchange, lowering oxygen levels and delaying respiration and ripening. As a result, OC fruits had lower weight loss, a slower increase in browning index and maturity index, and better visual and sensory scores than the CTR group. This dual strategy proved effective in extending shelf life while maintaining the fruit’s appearance, flavor, and nutritional value. It represents a sustainable and natural approach to enhancing the postharvest stability of lychee.

## 1. Introduction

The lychee (*Litchi chinensis* Sonn.), native to southern China and a member of the Sapindaceae family, is increasingly finding favorable conditions for cultivation in the Mediterranean region. In particular, the coastal areas of Sicily [1] have proven suitable for the cultivation of various tropical and subtropical species, primarily due to ongoing climate change [2].

This fruit is classified as non-climacteric and subtropical, and it therefore must be harvested at full ripeness to ensure its characteristic quality and desirable traits, including its bright and attractive appearance. Lychee is widely appreciated for its vibrant red skin, pleasant flavor, and rich nutritional content [3,4,5,6]. Among these traits, color vibrancy plays a crucial role in influencing consumer preference and purchasing decisions.

However, immediately after harvest, the fruit begins to lose its characteristic color due to oxidation of the pericarp [7,8]. The most significant postharvest issue is the fading of the bright red pericarp, which is often followed by pulp dehydration. This deterioration is accelerated under unfavorable postharvest conditions, such as high temperatures and low humidity, which promote anthocyanin oxidation and nutrient loss [9].

Two enzymes, peroxidase (POD) and polyphenol oxidase (PPO), play a key role in this process [10]. POD contributes to the peroxidation of membrane lipids and the formation of reactive oxygen species (ROS), while PPO catalyzes the oxidation of phenolic compounds, including anthocyanins, which are responsible for the fruit’s characteristic color [11]. The resulting pericarp browning is attributed to the excessive accumulation of H_2_O_2_ and O_2_, as well as the degradation of anthocyanins, ultimately causing both quantitative and qualitative losses of nutraceutical compounds [12].

To mitigate this issue, several studies have demonstrated that applying edible coatings can provide a protective barrier around the fruit surface, effectively reducing oxidative degradation and prolonging shelf life. As demonstrated in the study by Yang et al. [13], the application of a chitosan-based film enriched with natural pomelo extract significantly contributed to preserving the color of lychee fruit during storage [13,14]. Lychee’s phenolic compounds—including anthocyanins, flavonoids, tannins, and related metabolites—exhibit multiple bioactivities, such as antimicrobial, anti-inflammatory, and anticancer properties [1,2,3,4].

Numerous studies have shown that increased consumption of grains, fruits, and vegetables is correlated with a lower risk of developing chronic diseases [15,16,17]. This protective effect is largely attributed to naturally occurring antioxidants, such as vitamin C, tocopherols, carotenoids, polyphenols, and flavonoids, which help to counteract oxidative stress and free radical damage [18].

However, lychee suffers from significant postharvest losses, estimated between 20% and 50%, due to its high perishability and inadequate storage management [19]. Historically, fumigation with sulfur dioxide (SO_2_) was commonly employed to mitigate enzymatic browning. This treatment was effective in reducing the oxidation of phenolic compounds while preserving the fruit’s sensory attributes [20].

To date, fumigation with sulfur dioxide remains among the most broad-spectrum applications with immediate antimicrobial and antifungal action [21] on several fruits including grapes [22], bananas, lychees, apples [23], etc. However, the problem with this product, although effective, is determined by concentrations [24]. In fact, if high concentrations are used, adverse effects can be seen on both the treated products and the high risk to human health [25,26,27]. To remedy this problem, recent studies have applied several low-cost, naturally derived technologies to inhibit possible pathogens and enzymatic reactions such as the combination of oxalic acid and 1-methylcyclopropane (1-MCP) along with LDPE and HDPE on the physicochemical properties [28], bioactive compounds, antioxidants, and enzymatic activities of lychee fruits during cold storage [29].

To ensure consumer safety, chemical-based approaches must be phased out in favor of environmentally friendly and biodegradable alternatives, which not only help mitigate the emergence of drug-resistant pathogens [30] but also contribute to environmental sustainability. Among the most promising strategies are modified atmosphere packaging (MAP) [31,32,33], which helps maintain fruit quality and extend shelf life by creating a protective gaseous environment, and organic edible coatings, which reduce respiration rates, delay senescence, and provide an antimicrobial barrier that limits pathogen development while preserving fruit turgidity and freshness [30,34,35].

Within this context, growing attention is being directed toward the use of natural, biodegradable coatings to extend the postharvest life of highly perishable fruits such as lychee. Among these, olive oil-based coatings have emerged as a particularly promising alternative due to their unique physicochemical and bioactive properties [36,37].

Lipids contained in vegetable oils have gained increasing importance in food technology, not only for their nutritional value but also for their functional and structural contributions to food systems [34,35]. In addition to supplying energy and essential fatty acids, lipids enhance food texture, stability, and sensory attributes. Notably, lipid-based systems composed of liquid or semi-liquid vegetable oils can be structured into gel matrices, forming three-dimensional networks through the self-assembly of small gelling molecules. These architectures, ranging from liquid crystals to micelles and fibrillar networks, can create functional bilayers, making them ideal for edible coatings and controlled-release systems for bioactive compounds [36,37].

Extra virgin olive oil (EVOO) is particularly rich in monounsaturated fatty acids, predominantly oleic acid, and contains a wide spectrum of phenolic compounds, such as hydroxytyrosol, oleuropein, and oleocanthal, which exhibit strong antioxidant and antimicrobial properties. These bioactive molecules have been shown to mitigate oxidative stress and enzymatic browning by neutralizing reactive oxygen species (ROS) and inhibiting the activity of key enzymes, polyphenol oxidase (PPO) and peroxidase (POD), that are directly involved in anthocyanin degradation and pericarp darkening in lychee fruit [7,8,9,38].

In addition, the hydrophobic nature of olive oil contributes to moisture retention and establishes a physical barrier that limits oxygen permeability, thereby slowing down respiration and helping preserve the turgor and color of the fruit. The application of olive oil in edible coatings also aligns with the global effort to move away from chemical fumigants, such as sulfur dioxide (SO_2_), which, despite their effectiveness, pose potential health risks when used at high concentrations [39]. Consequently, incorporating olive oil into bio-based coating formulations offers a sustainable solution to reduce postharvest oxidative damage and enzymatic browning while simultaneously enhancing the nutritional quality and safety of lychee fruit.

The combined use of passive modified atmosphere packaging and organic coatings further amplifies these benefits by synergistically minimizing oxidative reactions and maintaining the fruit’s visual appeal and textural integrity [31]. While packaging slows the respiration rate, the organic coating provides an additional protective barrier against enzymatic browning and dehydration [40]. This integrated preservation strategy not only prolongs the postharvest quality of lychee but also supports its nutritional value and marketability over extended storage periods [41].

The objective of the present study was to investigate the effectiveness of an olive oil-based organic coating in mitigating enzymatic browning and oxidative stress in lychee fruit.

## 2. Materials and Methods

### 2.1. Collection of Lychee Fruits

Lychee fruits cultivar “Kwai Mai” was collected from “COLLURA” Farm, a local orchard located in Sant’Agata di Militello (ME), (38°03′30.0″ N 14°36′40.9″ E) in Sicily.

Two hundred fruits were harvested in September at commercial maturity according to Underhill and Wong [42], in which it is recommended that the ratio of °Brix to titratable acidity be around 40.

The color of the epicarp was used as an indicator to determine the ripeness of the fruit (pink–red). Fruits were collected for uniform size, shape, and color, and without visible defects.

Samples were previously sanitized with a 2% sodium hypochlorite solution for 20 min and then allowed to dry for submission to various trials [33].

### 2.2. Experimental Design

The 200 fruits collected were divided into two lots as follows:

- Control (CTR): 100 untreated fruits (control samples);
- Organic Coating (OC): 100 fruits were dipped in olive oil.

The olive oil was obtained by combining two Sicilian old varieties of olives, namely Verdello (60%) and Minuta (40%), thus creating a blend.

The study involved four bags of fruit (one for each day of analyses). The study was conducted with three replicates. After treatment, the fruits were allowed to dry for a few minutes and then stored in polyamide/polyethylene (PA/PE) bags (100 g of fruits × each bag), composed of 80% PA and 20% PE, with a thickness of 90 µm and a volume of 500 cm^3^, with an oxygen permeability of 47.6 cm^2^/(m^2^·day·atm) and a water vapor transmission rate of 3.9 g/(m^2^·day·atm). These properties affect the gas and moisture exchange between the fruit and the surrounding environment [43]. The bags were stored at a temperature of 5 ± 1 °C and a relative humidity of 90 ± 5% [43].

The analyses were performed at regular intervals, specifically on days 0 (as fresh product) and after 3, 6, and 9 days of cold storage. These analyses cover various aspects, including physical, chemical, microbiological, nutritional, pathological, and sensory attributes of the fruits.

### 2.3. Weight Loss

The weight loss of lychee fruits was verified on 5 packages (5 fruits each) for all CTR and OC treatments, respectively, and expressed in terms of percentage reduction from baseline using a precision digital scale to two decimal places (Mod. PCB 2000-1, Kern, Zanè, Italy).

### 2.4. Epicarp Color

A Minolta colorimeter, specifically the Chroma Meter CR-400 by Konica Minolta Sensing Inc. in Tokyo, Japan, was utilized to evaluate the color change in the fruit peel. The color space used for this evaluation is defined in terms of brightness (L* value), redness (a*), and yellowness (b*), following the CIELAB Lab* system.

Browning Index can be an important indicator of fruit ripeness or quality and may be used to assess changes in fruit condition over time calculated using the Equation (1) of Ruangchaket and Sajjaanantakul [44].

BI = [100 (x − 0.31)/0.17](1)

where x = (a* + 1.75L*)/(5.645L* + a* − 0.3012b*).

### 2.5. Firmness Factor

A texture analyzer, specifically the TA.XTplus, manufactured by Stable Microsystems, Ltd. in Surrey, UK, was used for this assessment. The texture (FF, N mm^−1^) analyzer was equipped with a load cell with a capacity of 25 kg. The testing procedure was conducted at a constant test speed of 0.5 mm s^−1^ on 5 fruit samples, and each survey was performed on three replicates.

### 2.6. Total Soluble Solids Content (TSSC) and Maturity Index (MI)

The juice was extracted from the lychee fruit using a centrifuge made by Ariete, a company based in Florence, Italy. It was used to separate the juice from the solid components of the lychee fruit. To determine the TSSC of the lychee juice, a digital refractometer made by Atago Co., Ltd. based in Tokyo, Japan was used.

To assess the degree of ripeness of the fruits, maturity index (MI) was determined through the following Equation (2) described by Peralta-Ruiz et al. [45]. It provides a numerical value that reflects the ratio between sweetness (TSSC) and acidity (TA) of the fruit, expressing the MI as an average with its standard error (SE).

MI = TSSC/TA(2)

### 2.7. Daily Pathological Surveys

Daily Pathological Surveys is a classification system that allows for a quick assessment of the contamination levels of the fruits based on the number of lesions present, with higher levels indicating more severe decay or contamination [46,47].

- No decay (N.D.): This category represents healthy fruits with no visible lesions or spots. It is assigned a contamination level of 0;
- Slight decay (S.D.): Fruits falling into this category have 1 to 4 lesions or spots. They are assigned a contamination level of 1;
- Moderate contamination (M.D.): Includes fruits with 5 to 10 lesions. They are assigned a contamination level of 2;
- High decay (H.D.): Fruits in this category have more than 10 lesions, and their surface is covered with spots. They are assigned the highest contamination level, which is 3.

### 2.8. Decay Index

The decay index (D.I.) is based on the methodology described by Morcia et al. [46], Barrera Bello et al. [47], and Perdones et al. [48] in relation to the evaluation of fruit decay due to the presence of fungi. This index (3) provides a numerical value that reflects the overall decay severity of the fruit under consideration, with higher values indicating more severe decay due to fungal contamination. It provides a quantitative assessment of the extent of fruit decay in the context of the study.

(3) $$D.I.=\frac{1n+2n+3n+4n\;}{N}$$

*n* is the number of fruits that have been classified at each previously indicated level of contamination. Levels of no decay (N.D.), mild decay (S.D.), moderate contamination (M.D.), and high decay (H.D.) are indicated.

*N* denotes the total number of fruits analyzed for each treatment within the specified range.

### 2.9. Visual Score and Marketability

The visual score for the lychee samples was determined by averaging the scores for color and aesthetic appearance. The calculation of the aesthetic quality was performed by creating a subjective scale with a maximum of 5 points. Specifically, 5 represents the maximum score where the aesthetic appearance of the fruit product is “excellent”, followed by 4 “good”, 3 “acceptable”, 2 “poor”, and finally 1 “very poor”, i.e., an inedible product. A score of 3 represents the limit of marketability for lychees [49].

The marketability of lychee fruit was evaluated according to the method of Saidi et al. [50] with some modifications. Descriptive quality characteristics were evaluated by assessing the degree of decay, surface defects, and shrinkage. Fruit quality was scored from 1 to 9, where 1–3 = unmarketable, 4 = fair, 5–7 = good, and 8–9 = excellent. The amount of marketable fruit was used as a parameter to calculate the percentage of marketable fruit during the shelf life.

### 2.10. Proximate Analysis

The proximate analyses of the flesh of lychee fruits were conducted to determine its chemical composition, including water content, total sugar content, and the levels of certain minerals such as potassium (K), sodium (Na), calcium (Ca), iron (Fe), and phosphorus (P) [51,52].

### 2.11. Microbiological Analysis

Microbiological analyses were conducted on the olive oil-based coating, the untreated (CTR), and coated (OC) lychee fruit samples. For each sample, 15 g of fruit and 10 mL of olive oil were first homogenized and then serially diluted in isotonic solution following the approach reported by Allegra et al. [53]. The resulting cell suspensions were plated on selective agar media (Table 1) to enumerate the microbial populations commonly associated with horticultural productions.

The detection of *Listeria monocytogenes* and *Salmonella* spp. was performed on 25 mL of olive oil-based coating and 25 g of CTR and OC lychee fruit samples in accordance with the ISO 11290-1 (2017) [54] and ISO 6579-1 (2017) [55] guidelines, respectively. All media were purchased from Oxoid (Basingstoke, UK). Plate counts on CTR and OC lychee fruit samples were conducted soon after production and after 3, 6, and 9 days of refrigerated storage. The results are expressed in Log CFU/mL or g and represent the average of three replicates.

### 2.12. Lychee Fruit Sensory Profile Evaluation

The sensory evaluation of lychee fruits was conducted with the involvement of ten semi-trained judges and the samples were presented to each panelist on a white Pyrex plate, coded with a three-digit code number. The evaluation utilized a hedonic liking test, which involved the judges rating various sensory attributes of the lychee fruit using a 9-point hedonic scale: 1 = “Extremely Disliked”; 5 = “Neither Liked nor Disliked”; 9 = “Extremely Liked”. The evaluation was performed considering 15 different sensory attributes of the lychee fruit: Flesh Color (FC); Firmness (C); Fruity Odor (FO); Exotic Fruit Odor (EXO); Off-Odor (OFO); Sweetness (S); Acidity (A); Juiciness (J); Astringency (AS); Pungency (PU); Fruit Flavor (FRF); Exotic Fruit Flavor (EXF); Alcohol Flavor (ALF); Off-Flavor (OFF); Overall Evaluation (OVE) [33].

### 2.13. Statistical Analysis

Prior to conducting parametric tests, the Shapiro–Wilk test was employed to ascertain the normality of the data across each treatment and storage day. A two-way analysis of variance (ANOVA) was conducted to evaluate the effects of treatment, storage time, and their interaction (TREAT × DAYS) on the measured parameters. In instances where substantial disparities were identified, Tukey’s post-hoc test was employed to undertake a comparative analysis of mean values at a significance level of *p* ≤ 0.01. For the analysis of the collected information, a factorial scheme with 5 replicates (*n* = 5), was employed, and statistical analysis was conducted using the software Minitab 17.1 (Minitab Inc., State College, PA, USA, 2013).

Data were presented as means ± standard deviations (SDs). To reduce the complexity of the multidimensional data on sugars, organic acids, and free amino acids and to facilitate their interpretation, we applied principal component analysis (PCA) using the R Studio (v. 4.2.1) package ggbiplot.

This technique made it possible to identify hidden patterns and trends in the data, providing a deeper understanding of the interactions between the factors analyzed and the chemical variables studied. In addition, to provide a visual representation of the relationships and inherent variations in the data, we generated heat maps using the R Studio (v. 4.2.1) heat map package ggplot2, which proved useful in highlighting correlations and significant differences.

## 3. Results and Discussion

### 3.1. Weight Loss

**Figure 1:**
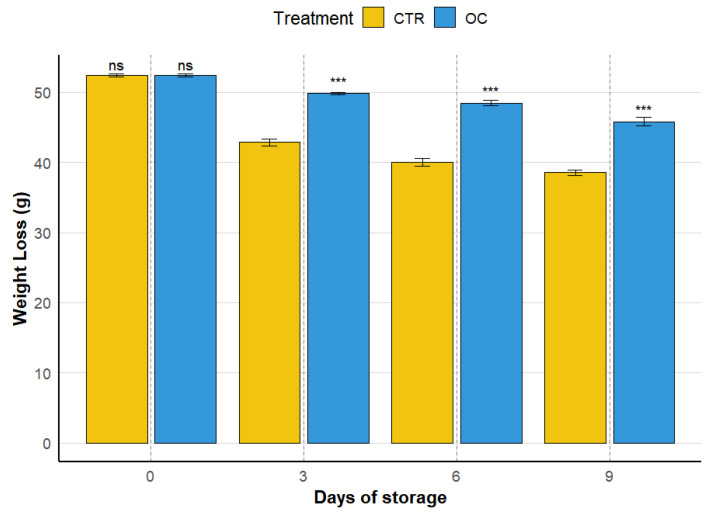
Effect of organic coating storage on weight loss of lychee fruit. Values are expressed as mean ± SD. Asterisks (***) above the histograms indicate significant differences (*p* < 0.01) between treatments at the same storage time; ns: not significant (Tukey’s test). CTR: control fruit; OC: treated sample with organic coating.

Weight loss in horticultural products is one of the main concerns in the postharvest sector as it serves as an indicator of carbon dioxide exchange, turgor pressure reduction, and increased respiration rates. The graph in Figure 1 illustrates the weight loss trends (expressed in grams) of OC and CTR samples over four storage intervals (0, 3, 6, and 9 days). As expected, at day 0, there were no differences between the two groups, since both started from the same initial weight.

The analysis of percentage weight loss reveals a clear divergence between the CTR and OC treatments, with significant differences over time. In the CTR group, the most substantial weight loss occurred in the initial period (days 0–3), showing an 18.3% reduction from baseline. This was followed by a slower decline: 6.8% between days 3 and 6 and 3.5% between days 6 and 9. This pattern suggests a progressive slowing of weight loss, possibly indicating a stabilization phase.

Conversely, the OC-treated fruit displayed a more moderated and consistent weight loss pattern. An initial 5% reduction was observed between days 0 and 3, followed by a uniform trend: 2.6% between days 3 and 6, and 5.5% between days 6 and 9. These data highlight a more controlled and gradual weight loss dynamic in the OC group compared to the CTR.

When comparing the two treatments, the CTR group experienced sharper and more abrupt weight loss in the early storage days, while the OC group maintained a more gradual and stable trend. These findings suggest that the OC coating may be more effective in minimizing water loss and preserving fruit integrity during storage. This physiological response is important, as excessive water loss is directly associated with accelerated quality deterioration in fruits [13,56].

Indeed, as highlighted in Figure 1, the weight loss in control (CTR) fruits was significantly higher compared to lychees treated with the organic coating (OC). The OC-coated fruits exhibited a more stable weight trend over time, likely due to the presence of polyethylene bags, which created a humid microenvironment around each fruit, effectively minimizing water vapor transmission [57].

In addition, the organic coating functioned as a semi-permeable barrier, reducing moisture loss and shielding the fruit from external environmental stress. This helped to lower respiration rates and delay the onset of senescence [58]. Maintaining high water content in the fruit tissues is crucial for preserving freshness and minimizing postharvest deterioration in horticultural products.

As confirmed by previous studies [48,49,50,51,52], edible films are capable of altering gas exchange by modifying the permeability to oxygen and carbon dioxide, thereby slowing down respiration and reducing water loss. In fact, weight loss typically occurs due to the vapor pressure gradient between the fruit’s internal environment and the surrounding atmosphere, which drives water migration and leads to gradual dehydration [59,60,61,62,63].

### 3.2. Epicarp Color

**Figure 2:**
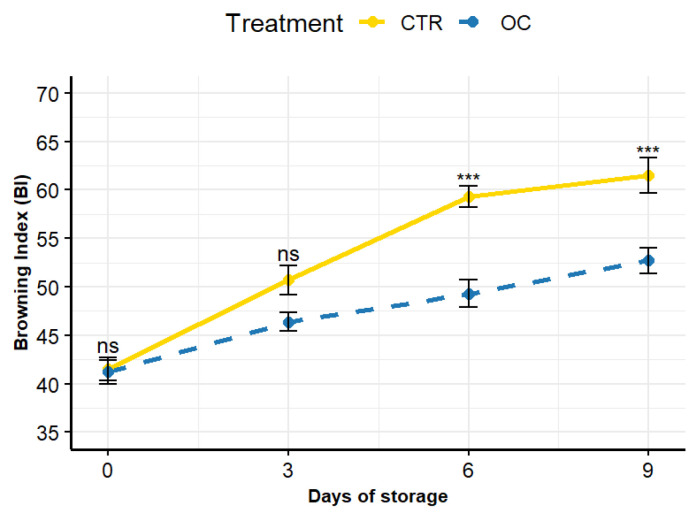
The evolution of peel Browning Index over a 9-day storage period. Lines represent mean values, while bars show standard deviations (*n* = 5). Asterisks (***) above the lines indicate significant differences (*p* < 0.01) between treatments at the same storage time; ns: not significant (Tukey’s test). CTR: control fruit; OC: treated sample with organic coating.

Biochemical parameters are indicative of the overall quality of the fruit, including the browning index, which is closely related to quality. As illustrated in Figure 2, an increase in browning was observed as the number of days of refrigerated storage increased. In fact, untreated fruit (CTR) exhibited a significant increase in browning (15.2%) compared to OC samples as early as the first three days of storage.

**Figure 3:**
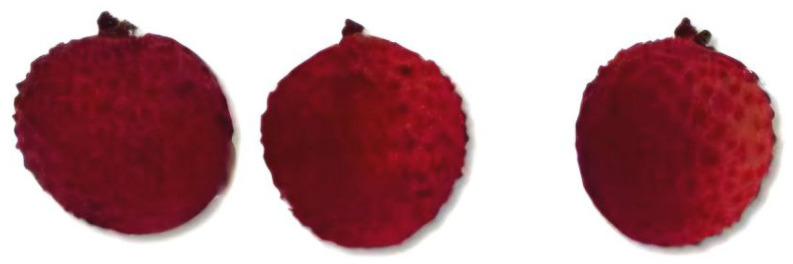
*Litchi chinensis* Sonn. on the last day of cold storage (day 9).

The browning index values in the CTRs exhibited a consistent upward trend, reaching 26.5% higher than in the coated lychees, which stabilized at approximately 49. On the final day of storage (day 9), the browning index of CTR was found to be 16.12% higher than that of OC (62 and 52, respectively) (Figure 3).

The lychee epicarp is particularly susceptible to browning due to the breakdown of cellular compartmentalization. Enzymes such as polyphenol oxidase (PPO) and peroxidase (POD) have been shown to play a key role in the degradation of polyphenols, which leads to the transformation of red pigments into brown compounds [12,64,65]. The structural and morphological integrity of the cell membrane is therefore essential for preserving fruit quality.

However, it is well established that during storage, lychee undergoes a series of physiological processes that ultimately lead to deterioration. The onset of senescence, and consequently the browning of the epicarp, is primarily associated with a decline in adenosine triphosphate (ATP) levels, triggering a cascade of metabolic events that accelerate fruit degradation [62].

Research has shown that ATP levels decrease rapidly after harvest compared to fruit at the climacteric stage. This reduction in ATP is a key factor contributing to the physiological deterioration of lychee [66,67,68]. Several studies [66,69] have highlighted that ATP plays a pivotal role in sustaining antioxidant defense mechanisms, supporting the synthesis of unsaturated fatty acids, and maintaining membrane integrity. The depletion of ATP leads to a series of structural damages, such as electrolyte leakage, which culminate in epicarp browning and the progression of senescence.

Phenolic compounds are therefore critical, not only due to their antioxidant capacity but also because of their interactions with enzymes [70,71]. These compounds have been shown to reduce metal ions that catalyze the formation of highly reactive species, such as hydroxyl radicals, which are detrimental to cellular membranes [36]. While reactive oxygen species (ROS) can be neutralized by the fruit’s antioxidant system, excessive ROS accumulation or compromised antioxidant activity can result in damage to the cytoplasm and plasma membranes, potentially causing hemolysis [60].

The application of coatings with dual functionality, benefiting both fruit preservation and human health, represents a sustainable and innovative approach. Studies have demonstrated that erythrocytes pre-treated with phenolic extracts from extra virgin olive oil exhibit significantly lower lipid peroxidation following H_2_O_2_ exposure [36]. Additionally, water retention has been recognized as one of the most crucial biochemical factors for maintaining the organoleptic properties of lychee during storage [10].

In the present study, the presence of phenolic compounds in the coating formulation was associated with reduced membrane damage and enhanced activity of key antioxidant enzymes, including superoxide dismutase (SOD), catalase (CAT), ascorbate peroxidase (APX), and glutathione reductase (GR). These enzymes contribute to the neutralization of ROS through both direct and indirect mechanisms [72,73]. Notably, the Browning Index (BI) parameter exhibited a strong correlation with fruit dehydration and consequent water loss. Specifically, it was found that the coating effectively reduced water loss, suggesting selective dehydration in the epicarp and limited moisture transfer between the arils and the outer peel [33,74]. Fruits treated with the coating showed a marked decrease in both respiration and ethylene production over time, highlighting the coating’s effectiveness in mitigating these physiological processes [75,76,77].

The clear separation between cell layers may be explained by the gradual thinning of the lychee epicarp during the ripening process, which typically reaches a thickness of 400–500 μm [75]. This thinning makes the epicarp increasingly vulnerable to dehydration and oxidative damage, potentially accounting for the higher decomposition index recorded in CTR samples.

Additionally, it has been reported that microcracks begin to develop in the epicarp as early as 20 days after fruit set [74], increasing the susceptibility of the peel to physiological stress. This fragility may explain the greater variability observed in the CTR group compared to the OC-treated fruits.

### 3.3. Firmness Factor

The utilization of polymer films, such as polyethylene, in the packaging of lychees is a prevalent postharvest practice. This method has been shown to enhance handling, reduce transportation and storage risks, and minimize moisture loss [78,79].

The application of polymer films not only safeguards the fruit from physical damage but also enhances its visual appeal. Specifically, polyethylene packaging creates a controlled atmosphere around the fruit, increasing CO_2_ levels and reducing O_2_ levels [42]. This modified environment effectively slows down respiration, water loss, and other metabolic processes [59,64].

**Figure 4:**
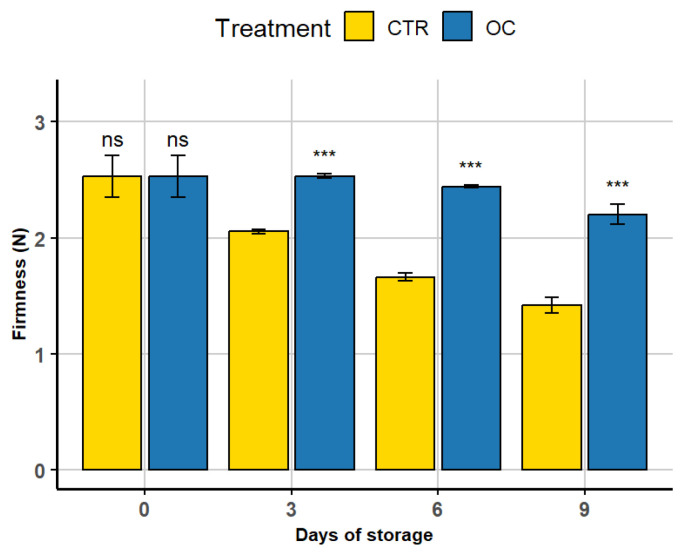
The decline in firmness of lychee fruits over a 9-day storage period is illustrated here. Each column represents the average firmness, while the error bars show the standard deviation (*n* = 5). Asterisks (***) above the histograms indicate significant differences (*p* < 0.01) between treatments at the same storage time; ns: not significant (Tukey’s test). CTR: control fruit; OC: treated sample with organic coating.

Texture, a key indicator of lychee freshness and consumer appeal, is associated with moisture retention, as weight loss frequently reflects fruit turgidity. At the start of the study (day 0), as represented in Figure 4, the firmness levels between untreated (CTR) and coated (OC) fruits exhibited no evident differences, with both measuring approximately 2.8 N.

On the third day, the coated fruits maintained their firmness, whereas the CTR fruits began to soften, reaching approximately 2 N. By the sixth day, the OC-treated samples preserved their texture with only a slight decrease, retaining approximately 73.33% of their initial firmness. By the ninth day, the firmness of the coated fruits was nearly twice that of the untreated ones. The progressive softening observed in the control group is commonly attributed to the breakdown of cell walls, primarily caused by the action of enzymes that degrade pectin and other structural components, ultimately leading to tissue degradation [80]. The marked reduction in firmness in untreated fruits during storage can be largely ascribed to water loss and an increase in soluble sugars. Moreover, the presence of antioxidant compounds in the coated samples may help preserve cellular integrity by inhibiting the enzymatic activities associated with softening [81]. Enzymes such as pectin methyl esterase (PME) and polygalacturonase (PG) play key roles in this process, as they catalyze the solubilization of pectin within the cell wall, leading to progressive loss of texture during fruit ripening [82,83]. This physiological response has also been documented in other postharvest fruits, including guava [81] and pear [67,69]. Furthermore, studies [81,84,85,86,87] have shown that the antimicrobial properties of olive oil help prevent infection and suppress respiration, thereby preserving fruit firmness. Elevated sucrose concentrations are known to disrupt osmotic balance between the external environment and the fruit’s cells, exacerbating dehydration and tissue collapse. As reported in the literature, this osmotic imbalance ultimately leads to cell water loss and surface shrinkage in lychee fruit [88].

### 3.4. PCA of Weight Loss (WL), Browning Index (BI), and Firmness Factor (FF)

**Figure 5:**
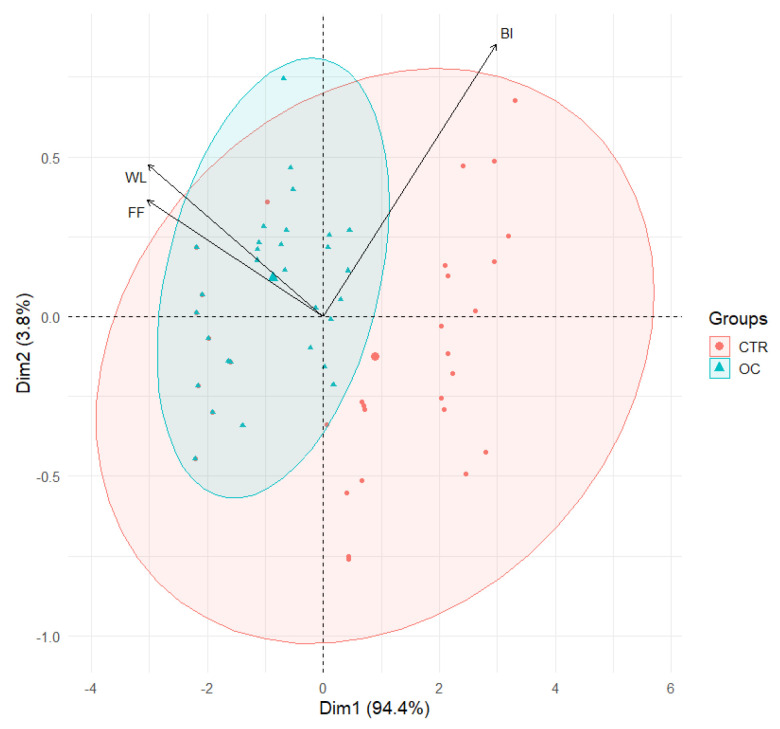
Principal component analysis (PCA) scores plot illustrating the distribution of browning index (BI), weight loss (WL), and firmness factor (FF) in control fruits (CTR) and OC treatment. The ellipses highlight the variability within each treatment group.

Principal component analysis (PCA) revealed significant associations between the browning index (BI), weight loss (WL), and firmness (FF), as illustrated in Figure 5, thereby providing deeper insight into the dynamics of fruit quality across different treatments. A clear positive correlation was observed between BI and WL, indicating that fruits experiencing greater weight loss tend to exhibit more pronounced browning. This suggests that dehydration may promote browning by accelerating oxidative processes. This relationship appears to be stronger in CTR samples compared to OC-treated fruits, implying that CTR fruits are more vulnerable to browning as moisture is lost.

Furthermore, the analysis showed an inverse correlation between BI and firmness, with higher browning levels corresponding to reduced texture quality. These findings are consistent with those reported in previous studies [18,71,73].

Fruits with a softer texture, characterized by a reduced firmness, exhibit a higher propensity for browning, suggesting that structural degradation of the fruit contributes to increased browning [67]. This correlation is particularly evident in CTR samples, which exhibit greater variability in texture and are often linked to higher levels of browning [41,89].

Conversely, the application of organic coating (OC) has been observed to promote a more consistent browning response over time, potentially due to the maintenance of a more uniform texture.

The correlation between weight reduction and diminished texture is well documented. As fruits undergo a reduction in weight, they concurrently experience a decline in turgor pressure, leading to a diminution in texture [90]. The effect is more pronounced in CTR samples, where weight loss is associated with a substantial decrease in texture, indicating a more rapid decline in quality.

Conversely, the application of OC exhibited a comparatively less pronounced impact, suggesting that it may be more efficacious in preserving the physical integrity of the fruit.

A comparative analysis of the two treatments reveals that OC demonstrates superior efficacy in preserving fruit quality, maintaining low browning levels and texture with minimal weight loss.

The PCA graph (Figure 5) demonstrates that the OC samples are grouped more closely together, with reduced variability, especially along the PC2 axis. This finding suggests that the application of OC treatment results in more uniform and predictable outcomes regarding browning, weight loss, and texture.

Conversely, the CTR samples exhibited increased dispersion across both PCA components, indicative of heightened variability in these quality parameters. This finding suggests that the efficacy of CTR in mitigating fruit quality deterioration may be diminished.

The relationships between BI, WL, and F indicate that the quality of CTR (untreated) fruit is less predictable, with greater susceptibility to browning and physical degradation.

The PCA analysis provides a clear illustration of the significant advantage offered by OC treatment in preserving fruit quality in comparison to CTR.

### 3.5. Total Soluble Solid Content (TSSC) and Maturity Index (MI)

**Figure 6:**
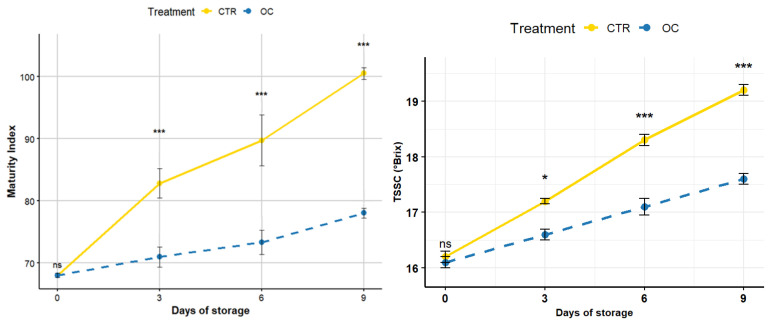
Evolution of maturity index (MI) of lychee during 9 days of storage for CTR (control) and OC treatments. Lines indicate averages, while bars represent standard deviation (*n* = 5). Asterisks indicate statistically significant differences between treatments at each storage time. *: *p* ≤ 0.05, ***: *p* ≤ 0.001; ns: not significant (Tukey’s test). CTR: control fruit; OC: treated sample with organic coating.

The results of maturity index (MI) of lychee during the 9-day storage period are graphically represented in Figure 6.

The CTR sample (solid line) shows a significant increase in MI during the storage period, suggesting a greater acceleration of the maturation process.

The OC sample (dashed line), on the other hand, shows a more gradual increase in MI, indicating that this treatment is more effective in slowing down the ripening process of the fruit, as revealed in other studies [59,72,91]. This was also observed in guava, where the olive oil coating acted as a semi-permeable barrier, slowing down metabolism and respiration [85]. Similar results have also been obtained in other studies, for example, in sweet orange [92] and carambola coated with a combination of sodium alginate and olive oil and stored for 16 days at 25 ± 5 °C [93].

In terms of sugars, starch is converted into simple sugars such as glucose and fructose [82,94]. However, thanks to the protection of the film on the surface of the lychees, it was possible to act by creating a selective barrier, alternating the internal atmosphere and slowing down the respiration of the fruit [56,57,95].

Similar results, in which sugars were preserved during storage, were obtained with strawberries covered with chitosan and olive oil residues [8,43,44].

The result suggests that the OC treatment with coating is effective in delaying the ripening process compared to CTR, helping to maintain product quality during cold storage. OC is a valid strategy for extending shelf life, keeping the product in better condition and reducing the risk of premature spoilage [96,97].

### 3.6. Daily Pathological Surveys and Decay Index (DI)

Analysis of the decay index (DI) trend during the cold period shows significant differences between the CTR and OC treatments, with OC showing a greater ability to delay decay than CTR. On day 0, both treatments show a decay index of 0, indicating no initial deterioration.

**Figure 7:**
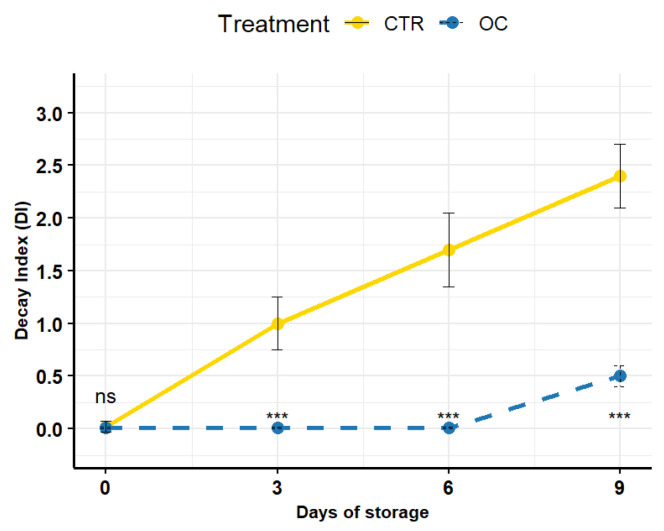
Decay index (D.I.) rated from 1 to 4 on lychee fruit measured for 9 days post-treatment in cold storage at 5 ± 1 °C. Lines indicate averages, while bars represent standard deviation (*n* = 5). Asterisks indicate statistically significant differences between treatments at each storage time. ***: *p* ≤ 0.001; ns: not significant (Tukey’s test). CTR: control fruit; OC: treated sample with organic coating.

As shown in Figure 7, a clear discrepancy was observed on the third day: the decay index for the CTR treatment reached 1.0, while the OC treatment remained at 0.5. This absolute difference of 0.5 indicates that decay in CTR samples was twice that observed in OC, corresponding to a 100% increase. These findings suggest that the OC treatment may begin to slow the onset of decay from the earliest stages of storage.

By the sixth day, the decay index continued to rise in both treatments, reaching 1.5 for CTR and 0.7 for OC. The absolute difference widened to 0.8, further confirming that decay progression was more pronounced in CTR, while OC maintained a slower, more controlled increase.

On the ninth day, the CTR group reached a maximum decay index of 2.5, whereas OC peaked at 1.0, resulting in an absolute difference of 1.5. This may be attributed to the coating’s ability to seal microfractures that commonly form during handling and storage. Such microfractures often serve as entry points for pathogenic microorganisms and promote fungal colonization [36,59,60,61,91].

These results underscore the effectiveness of the OC treatment in limiting decomposition—particularly visible decay—by delaying pathogen development and disease spread [62,98].

### 3.7. Visual Score and Marketability

**Figure 8:**
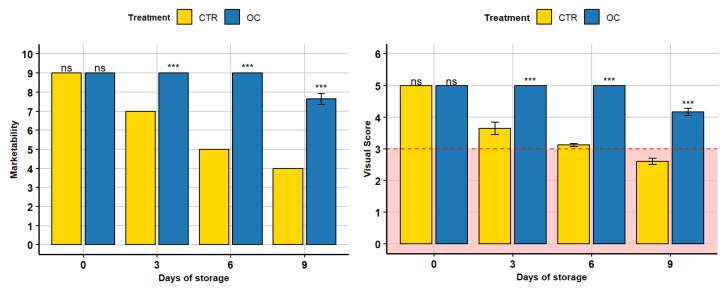
Percentage marketability (on the left) and visual score (on the right) of litchi fruits stored at 5 ± 1 °C for up to 9 days, comparing uncoated (CTR) and coated (OC) treatments. Values are presented as mean ± standard deviation (*n* = 5). Asterisks indicate statistically significant differences between treatments at each storage time. ***: *p* ≤ 0.001; ns: not significant (Tukey’s test). For visual score evaluation, the following scale was used: 5 = very good, 4 = good, 3 = fair (marketability threshold), 2 = poor (edibility threshold), 1 = very poor (inedible). Pink area indicates the marketability threshold.

In relation to the aesthetic appearance and marketability of lychees, the visual score and % of marketability provides a rating and assessment of product acceptability. Indeed, in Figure 8, the visual score for CTR fruits steadily decreased over time, with a significant drop by day 9 during cold storage compared to treated fruits.

In contrast, coated lychees enriched with oil had a significantly higher score, remaining within the range of marketability and edibility until the last storage day (day 9), as defined by the black line at Level 3, below which fruits lose their marketability [50,56,99]. The red dashed line and orange shaded area represent the minimum quality threshold, within which untreated fruits fall.

### 3.8. PCA Visual Score, Marketaability, and Decay Index

**Figure 9:**
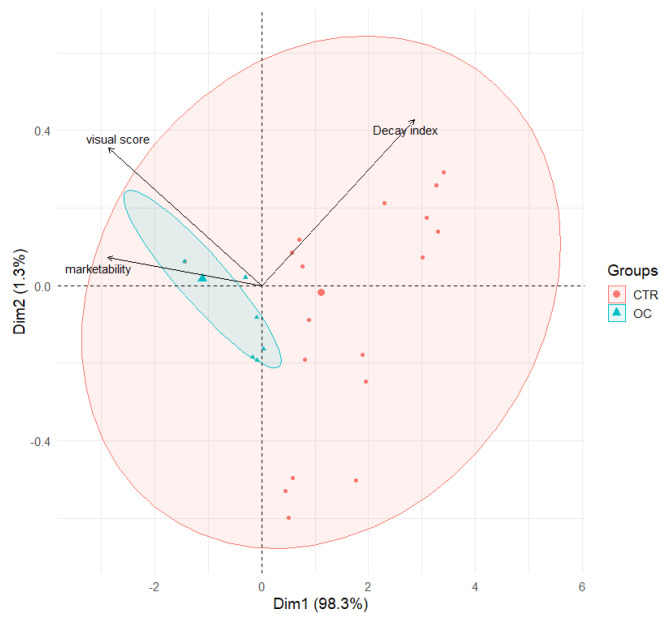
Principal component analysis (PCA) scores showing decay index, visual score, and marketability for CTR and OC treatments. The ellipses indicate variability within each group.

The PCA plot in Figure 9 presents an analysis of three key variables: decay index, marketability, and visual score. Principal component analysis (PCA) is particularly valuable for reducing the dimensionality of multivariate data and visually representing the relationships between samples, thereby enabling effective assessment of similarities and differences across treatment groups [100,101].

The x-axis (PC1) captures 97.4% of the total variance in the dataset, indicating that nearly all meaningful variation among the samples is explained by this component. This makes PC1 the principal axis differentiating the treatments based on the three variables. The y-axis (PC2) accounts for only 2.1% of the variance, suggesting it contributes minimally to the separation of groups [102,103].

Each point on the plot represents an individual sample, corresponding to either the CTR or OC treatment. The ellipses surrounding the data points represent the distribution and variability within each treatment group, outlining the region where most of the samples are concentrated.

The ellipse for the CTR group is broader, indicating higher internal variability and a more heterogeneous response in terms of decay index, marketability, and visual score. In contrast, the ellipse for the OC group is more compact, reflecting greater consistency and a more uniform treatment effect.

The distribution of points further underscores the separation between treatments: CTR samples are dispersed primarily on the right side of the plot, with substantial spread along the PC1 axis, highlighting inconsistent outcomes. Meanwhile, OC samples cluster tightly on the left side, demonstrating less variability and a more stable response to the treatment.

PC1 clearly distinguishes between the two groups, with CTR samples exhibiting higher values and OC samples lower values along this axis. This indicates that PC1 encapsulates a weighted combination of the three variables that effectively discriminates between the treatments. PC2, on the other hand, contributes little to the overall differentiation, reaffirming that PC1 is the dominant axis of variation.

Overall, Figure 9 demonstrates that the OC treatment leads to a more homogeneous and predictable response, as evidenced by the tighter clustering and reduced variability. The strong explanatory power of PC1 (97.4%) suggests that the decay index, marketability, and visual score are highly interrelated in differentiating the treatments. In contrast, the CTR group exhibits greater dispersion, indicating a less consistent effect on fruit quality. Therefore, the PCA provides robust evidence that the OC treatment is more effective in maintaining consistent and desirable quality attributes in lychee fruits compared to the control.

### 3.9. Heatmap of Quality Parameters

**Figure 10:**
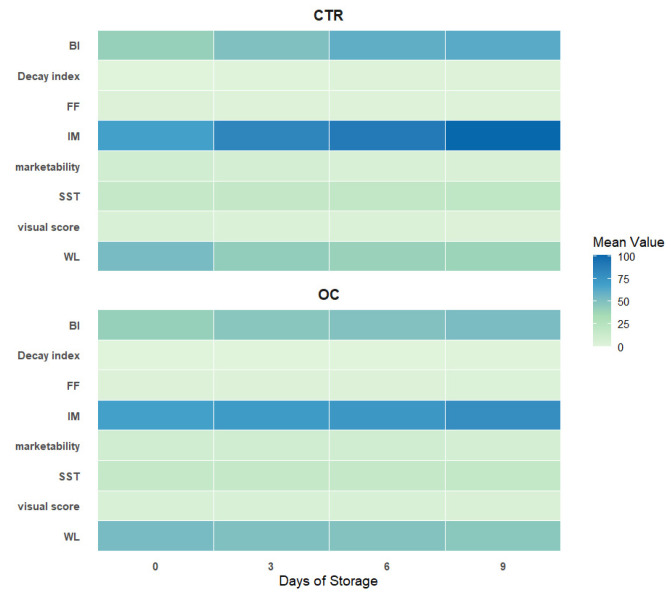
The heatmap shows the correlation matrix between all numerical variables in the dataset. Each cell represents the correlation between two variables, with the numerical values ranging from −1 to 1: 1 indicates perfect positive correlation, if one variable increases, the other always increases as well; −1 indicates perfect negative correlation, if one variable increases, the other always decreases; 0 indicates no correlation between the variables. The coloring of the cells helps visualize the strength and direction of the correlation: Pale green (low values) to deep blue (high values).

The analytical framework illustrated in Figure 10 provides a comprehensive depiction of the temporal evolution of key quality parameters in lychee fruits over a nine-day cold storage period. This analysis compares untreated samples (CTR) with those treated using an organic olive oil-based coating (OC).

The inclusion of a heat map enables an intuitive visualization of the mean values of each parameter at specific time points. This is achieved through a natural color gradient, ranging from pale green (low values) to deep blue (high values), which facilitates immediate interpretation of changes and intensities in quality attributes.

In the CTR group, the maturity index (MI) showed a marked increase by day 9, indicating a rapid ripening process. This change was accompanied by a progressive decrease in weight (WL), an increase in the browning index (BI), and a steady rise in the decay index, all of which point to physiological and microbial degradation. Additionally, a gradual loss in firmness (FF) was observed, suggesting the breakdown of tissue structure and mechanical integrity. Consumer-relevant traits such as visual score and marketability declined sharply throughout storage, reflecting a rapid deterioration in both aesthetic and commercial value.

By contrast, fruits treated with the OC coating exhibited markedly improved stability. Although MI still increased, it remained more constant over time. Parameters such as FF, BI, WL, and decay index consistently remained lower than those observed in CTR fruits, indicating the coating’s effectiveness in mitigating senescence, water loss, and structural breakdown.

The correlation matrix accompanying the heat map further highlights significant interdependencies among key quality indicators. Notably, visual score and marketability showed an almost perfect positive correlation (r = 0.98), underscoring the critical influence of appearance on consumer purchasing behavior. In contrast, the decay index demonstrated strong negative correlations with both visual score (r = –0.96) and marketability (r = –0.98), confirming that even minor visual defects can have a substantial impact on perceived quality and commercial appeal [104,105,106].

### 3.10. Proximate Analysis

A comparison between the control sample (CTR) and the treated sample (OC) with respect to the retention of key minerals (K, Na, Ca, P, Fe) throughout the storage period (Table 2) reveals marked differences in their preservation dynamics.

Regarding potassium (K), the CTR group exhibited a significant decline over time, particularly between day 6 and day 9. In contrast, OC-treated fruits showed a significantly enhanced ability to retain K levels, maintaining higher concentrations throughout storage [107]. The divergence between the two treatments became especially apparent on days 6 and 9, with OC outperforming CTR in preserving K content [108].

In the case of sodium (Na), both groups maintained relatively stable levels throughout the storage period, with only a modest overall decline and no substantial difference between CTR and OC [109].

Calcium (Ca) levels declined notably in the CTR group, particularly by day 9. Conversely, OC-treated samples demonstrated greater stability, retaining higher Ca concentrations compared to CTR. A significant increase in Ca content was observed in the OC group at day 9, suggesting that the coating played a protective role in mineral preservation.

For phosphorus (P), a gradual decline was recorded in the CTR group throughout the study [110], whereas OC-treated fruits consistently maintained higher P levels at each time point [106], suggesting superior phosphorus retention.

With respect to iron (Fe), the CTR group showed a sharp reduction immediately after day 0, maintaining consistently low levels for the remainder of the storage period. In contrast, the OC group exhibited significantly greater Fe retention, indicating better mineral preservation under coated conditions.

Collectively, these results suggest that OC treatment is markedly more effective in preserving mineral content, particularly K, Ca, and P, when compared to the untreated control. These findings are consistent with those of previous studies [16,33], which have demonstrated that organic coatings provide a protective barrier that limits nutrient degradation.

This mineral retention capability not only supports the use of OC in postharvest preservation strategies but also highlights its potential in maintaining the nutritional integrity of fresh produce during storage.

### 3.11. Microbiological Analysis

The microbiological analysis carried out by a culture-dependent approach on olive oil-based coating, untreated (CTR), and coated (OC) lychee fruit samples aimed to identify microbial populations that affect the quality, hygiene, and safety of horticultural products during refrigerated storage [111,112]. The microbiological analysis of the olive oil-based coating showed no presence of bacteria, yeasts, or molds, confirming its effectiveness as a natural edible coating for postharvest treatments. During the entire storage period, none of the lychee fruit samples showed presence of pseudomonads and yeast, which are responsible of microbial spoilage of fruit commodities [113], nor *E. coli*, CPS, *L. monocytogenes*, and *Salmonella* spp., which are known to cause foodborne diseases [114,115,116,117].

These findings align with those observed by Passafiume et al. [33] for lychee fruits stored in both passive and MAP packaging. As expected, only TMM and moulds were detected on the lychee fruits, which are the main causes of microbial decay during their postharvest life [118,119]. As reported in Table 3, statistically significant differences between the trials were observed after 6 days of storage, when TMM and moulds appeared at around 10^4^ CFU g^−1^ in CTR and 10^3^ CFU g^−1^ in OC fruit samples.

The concentrations of these microorganisms increased at 9 days of refrigerated storage, but the OC-treated fruit showed final levels of about 1 log cycle lower than those estimated for CTR production. This result can be attributed to the protective olive oil-based coating, which act as a barrier on the fruit surface, limiting microbiological decay [57,115,116].

### 3.12. Lychee Fruit Sensory Profile Evaluation

**Figure 11:**
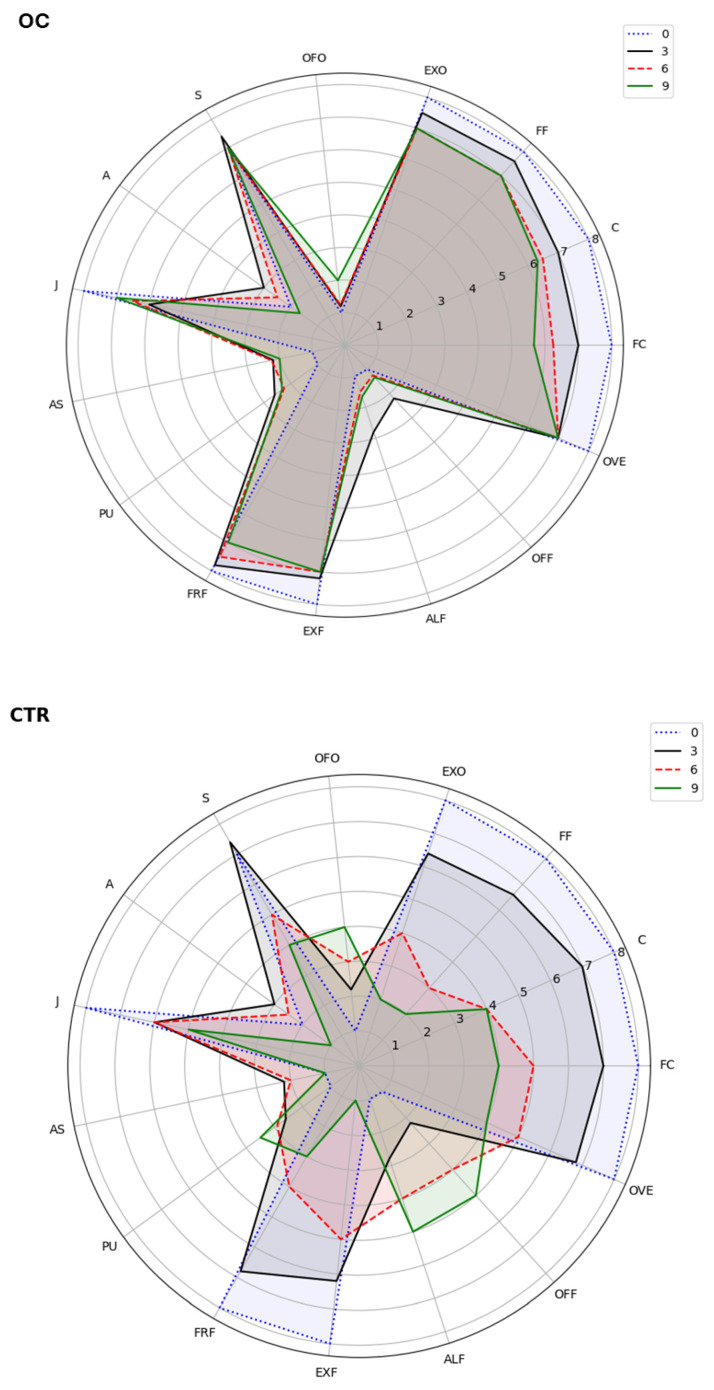
Sensory analysis of lychee fruits treated (OC) and untreated (CTR), during cold storage at 5 ± 1 °C after 0; 3; 6; 9 days.

In the sensory evaluation of lychee fruits during cold storage (Figure 11), panelists reported significant differences between the untreated (CTR) and the coated (OC) samples across the storage period. By day 9, CTR fruits exhibited noticeably higher scores for off-flavors and undesirable attributes compared to OC samples, indicating that the olive oil coating treatment more effectively preserved sensory quality.

Marked differences emerged in key sensory parameters. For example, flesh color (FC) and firmness (C) were better maintained in OC-treated fruits, with scores approximately 1.5 times higher than those of CTR at the end of the storage period. This underscores the effectiveness of the OC treatment in preventing browning and preserving textural integrity. Moreover, OC fruits received higher ratings for exotic fruit odor (EXO) and fruity flavor (FRF), approximately 1.3 times greater than CTR by day 9, demonstrating that the coating helped preserve the fresh, appealing sensory profile associated with high-quality lychee [33].

Overall, panelists expressed a clear preference for the OC-treated fruits by the end of the storage period, noting that the natural flavors were well-preserved and no off-flavors were detected [120]. This aligns with the coating’s known ability to reduce biochemical degradation, retain sweetness, and minimize moisture loss, factors critical for maintaining overall fruit marketability. The olive oil-based coating acted as a semi-permeable barrier, slowing the degradation of volatile compounds and preserving both texture and appearance, consistent with prior findings on the efficacy of edible coatings [121].

According to several studies [41,122,123], coatings made with olive oil or essential oils act as selective barriers to oxygen, moisture, and carbon dioxide, thereby reducing respiration and delaying ripening while maintaining sugar and acid levels that are acceptable to consumers [43,124]. Notably, olive oil’s protective action stems from its antioxidant and antimicrobial activities, attributed to phenolic compounds such as tyrosol, hydroxytyrosol, and ligstroside aglycone, as highlighted in the literature [91,125,126,127]. These bioactive molecules not only extend shelf life but also contribute health benefits, including anti-inflammatory, antiviral, and cardioprotective effects, as extensively documented [59,60,61,91,128].

## 4. Conclusions

The proposed olive oil-based coating, when combined with PA/PE passive atmosphere packaging, has been shown to be highly effective in preserving the visual, structural and microbiological quality of fresh lychees during cold storage. In contrast to conventional coatings derived from chitosan, alginate, or CMC, which frequently necessitate chemical modifications, crosslinkers, or additives, the olive oil-based system constitutes a single-component, naturally bioactive solution of plant origin. The inherent properties of this substance, namely its barrier, antioxidant, and antimicrobial characteristics, obviate the necessity for formulation steps, thus rendering it optimal for cost-effective, clean-label applications. In addition, olive oil has received GRAS certification, is allergen-free, and is compatible with vegan dietary requirements, thus surpassing the significant limitations of animal-derived polymers such as chitosan. The lipidic nature of the substance has been demonstrated to offer effective control of moisture loss and enzymatic browning, with no requirement for drying or post-processing steps. The system is also fully compatible with existing postharvest workflows, and its gel-like behavior suggests promising potential for the future development of structured organogels. This approach is a sustainable, inclusive and easily implementable strategy for fruit preservation. It is particularly relevant for local or low-input food systems.

## Figures and Tables

**Table 1:** Selective agar media and growth conditions.

Microorganisms	Media	Growth Conditions
Total mesophilic microorganisms	Plate Count Agar	3 d at 30 °C
Pseudomonadaceae	*Pseudomonas* Agar Base	2 d at 25 °C
Enterobacteriaceae	Violet Red Bile Glucose Agar	1 d at 37 °C
Coagulase-positive staphylococci	Baird Parker Agar	1 d at 37 °C
*Escherichia coli*	Chromogenic Medium	1 d at 37 °C
Unicellular fungi (yeasts)	Yeast Peptone Dextrose Agar	2 d at 30 °C
Filamentous fungi (moulds)	Potato Dextrose Agar	7 d at 25 °C

**Table 2:** Levels of mineral elements (potassium, sodium, calcium, phosphorus, iron) evaluated over the storage duration. The values are expressed as averages ± standard deviation (*n* = 5). Above each value, lowercase letters indicate differences (*p* < 0.01) between treatments at the same storage time, while uppercase letters denote differences (*p* < 0.01) over time within the same treatment (Tukey’s test). CTR: control fruit; OC: treated sample with organic coating.

		Days of Storage			
	Treatments	0	3	6	9
K (mg/100 g FW)	CTR	39.12 ± 0.34 ^aA^	33.75 ± 0.22 ^bA^	28.57 ± 0.23 ^bB^	18.4 5± 0.56 ^bB^
	OC	39.12 ± 0.34 ^aA^	36.45 ± 0.14 ^aA^	30.11 ± 0.16 ^aA^	24.36 ± 0.74 ^aA^
Na (mg/100 g FW)	CTR	10.54 ± 0.27 ^aA^	7.88 ± 0.22 ^aA^	7.54 ± 0.48 ^aA^	7.10 ± 0.65 ^aA^
	OC	10.54 ± 0.27 ^aA^	8.12 ± 0.73 ^aA^	7.63 ± 0.29 ^aA^	7.32 ± 0.17 ^aA^
Ca (mg/100 g FW)	CTR	15.28 ± 0.71 ^aA^	13.46 ± 0.28 ^aA^	12.15 ± 0.24 ^aA^	9.24 ± 0.30 ^bB^
	OC	15.28 ± 0.71 ^aA^	14.22 ± 0.62 ^aA^	13.37 ± 0.53 ^aA^	12.89 ± 0.10 ^aA^
P (mg/100 g FW)	CTR	45.20 ± 0.10 ^aA^	35.17 ± 0.12 ^bA^	24.57 ± 0.13 ^cB^	22.43 ± 0.24 ^dB^
	OC	45.20 ± 0.10 ^aA^	43.28 ± 0.15 ^aA^	40.28 ± 0.27 ^aA^	30.28 ± 0.14 ^aA^
Fe (mg/100 g FW)	CTR	0.30 ± 0.34 ^aA^	0.12 ± 0.11 ^bB^	0.09 ± 0.12 ^bB^	0.09 ± 0.13 ^bB^
	OC	0.30 ± 0.34 ^aA^	0.25 ± 0.26 ^aA^	0.20 ± 0.16 ^aA^	0.13 ± 0.17 ^aA^

**Table 3:** Microbial loads of lychee fruit samples, for both untreated (CTR) and coated (OC) treatments, during cold storage at 5 ± 1 °C over 9 days.

Storage Time	Samples	Microbial Loads	
		TMM	Moulds
0 days	CTR	<2	<2
	OC	<2	<2
3 days	CTR	<2	<2
	OC	<2	<2
6 days	CTR	3.98 ± 0.08 ^b^	3.80 ± 0.19 ^b^
	OC	3.08 ± 0.10 ^a^	2.98 ± 0.11 ^a^
9 days	CTR	5.10 ± 0.12 ^b^	5.02 ± 0.09 ^b^
	OC	4.25 ± 0.12 ^a^	4.30 ± 0.10 ^a^

Units are log CFU g^−1^. Results indicate mean values ± S.D. of three plate counts. Superscript letters indicate differences (*p* < 0.01) between treatments at the same storage time (Tukey’s test). TMM: total mesophilic microorganisms.

## Data Availability

The data presented in this study are available on request from the corresponding author.
